# SIRT6 in Cancer: Mechanistic Insights into Its Dual Roles in Cancer Biology and Implications for Precision Therapeutic Development

**DOI:** 10.3390/biom15121655

**Published:** 2025-11-26

**Authors:** Yanqi Feng, Zhuoyan Han, Kunrui Zhu, Yuelin Han, Xiangtian Xiao, Jie Tong, Yiming Li, Shu Xia

**Affiliations:** Department of Oncology, Tongji Hospital, Tongji Medical College of Huazhong University of Science and Technology, 1095 Jie Fang Avenue, Wuhan 430030, China

**Keywords:** SIRT6, metabolic reprogramming, context-dependent duality, precision oncology

## Abstract

Sirtuin 6 (SIRT6), a (Nicotinamide adenine dinucleotide) NAD^+^-dependent deacylase and mono- (adenosine diphosphate) ADP-ribosyltransferase, is increasingly recognized as a pivotal regulator of genomic stability, metabolic reprogramming, and epigenetic remodeling. This review synthesizes current evidence on the dual roles of SIRT6 in cancer, highlighting its context-dependent functions as both a tumor suppressor and promoter across various malignancies. We detail its involvement in DNA damage sensing, repair coordination, glycolytic regulation, and chromatin modification, and discuss how these mechanisms contribute to tumor initiation, progression, and therapy resistance. Emerging therapeutic strategies targeting SIRT6, including small-molecule modulators, genetic interventions, and combination therapies, are critically evaluated. Our analysis underscores the necessity for context-specific therapeutic targeting, and pharmacological modulation of SIRT6 represents a promising avenue for precision oncology.

## 1. Introduction

The mammalian Sirtuin family (SIRT1-7) comprises NAD^+^-dependent class III histone deacetylases that are highly conserved across prokaryotes and eukaryotes and that participate in essential cellular processes such as inflammation; metabolism; oxidative stress; and apoptosis. Each of the seven sirtuins displays distinct subcellular localization, catalytic activity, and substrate specificity, enabling them to exert dual roles in various pathological processes such as cancer; cardiovascular diseases; and respiratory disorders [1,2]. Recent studies have shown that abnormal expression of SIRT is present in almost all types of cancer, and its mechanism is related to cancer metabolism; genomic stability; and the tumor microenvironment [3,4,5].

Similar to other members of the SIRT family, SIRT6 possesses a conserved NAD^+^-dependent catalytic core, although its active-site architecture exhibits subtle structural distinctions [6]. Beyond its canonical deacetylase function, SIRT6 also displays deacylase and mono-ADP-ribosyltransferase activities [7,8]. SIRT6 is predominantly a chromatin-associated nuclear protein that modulates gene expression through chromatin remodeling, thereby serving as a key regulator of chromatin dynamics [9,10]. SIRT6 influences cellular physiological and pathological processes through multiple mechanisms, such as DNA repair, which plays a critical role in maintaining genomic stability. Furthermore, SIRT6 regulates glucose and lipid metabolism and is intricately linked to aging and cellular stress responses [11,12,13,14].

SIRT6 also plays a complex role in cancer progression. It acts as either a tumor suppressor or tumor promoter depending on the cancer type and cellular context [15]. In certain malignancies, SIRT6 suppresses tumor growth by regulating key processes such as DNA repair, apoptosis, and autophagy. Whereas in other contexts, SIRT6 overexpression correlates with a more aggressive phenotype and poor prognosis [16]. Targeting SIRT6 therapeutically could offer novel avenues for cancer treatment, with the potential to modulate its activity through small molecules or gene therapy, offering hope for personalized cancer therapies.

### 1.1. SIRT6 Is a DNA Damage Sensor and Guarder of Genomic Stability

**Figure 1:**
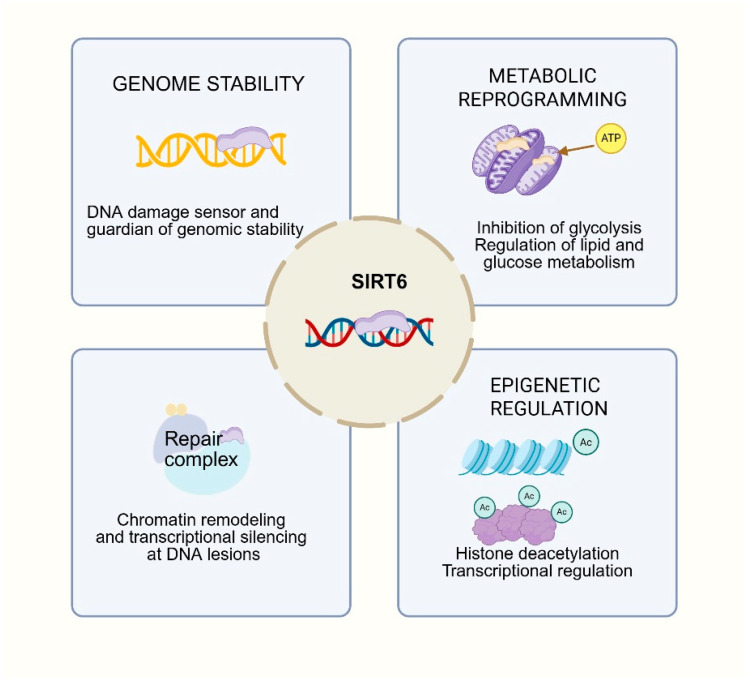
The function of SIRT6 in cancer progression.

A distinctive feature that sets SIRT6 apart from other sirtuins is its unique ability to function as an early DNA double-strand break (DSB) sensor, directly binding damaged DNA via a tunnel-like structural domain (Figure 1). This DNA-binding property is fundamental to stimulating its catalytic activity and orchestrating downstream repair processes [17,18]. Upon DSB recognition, SIRT6 recruits and activates ATM kinase, thereby facilitating phosphorylation of H2AX and promoting the assembly of repair complexes essential for homologous recombination (HR) and non-homologous end joining (NHEJ) [19].

Beyond its direct sensing role, SIRT6 actively remodels chromatin at DNA lesions. It cooperates with CHD4 to relax nucleosomes, enabling efficient access of repair proteins [20]. Moreover, SIRT6 deacetylates RNA-binding proteins such as hnRNPA2B1, enforcing local transcriptional silencing at DSB sites [21]. This prevents transcription–replication conflicts, a well-recognized source of genome instability, and its loss results in inaccurate repair.

SIRT6 further safeguards chromosome segregation during mitosis. Through reciprocal regulation with the anaphase-promoting complex/cyclosome (APC/C), SIRT6 undergoes ubiquitin-proteasome degradation while simultaneously deacetylating CDH1 to modulate APC/C activity [22]. Disruption of this regulatory axis results in centrosome amplification, chromosome missegregation, and aneuploidy, which directly links SIRT6 loss to chromosomal instability.

Compelling pharmacological and genetic evidence further demonstrates its role as a genome guardian. The small-molecule activator MDL-800 enhances both NHEJ and base excision repair, which improve repair capacity in aged cells and boost iPSC reprogramming efficiency [23]. In humans, rare centenarian SIRT6 variants (N308K/A313S) exhibit enhanced interaction with Lamin A, increased mono-ADP-ribosyltransferase activity, stronger retrotransposon suppression, and improved cancer cell killing compared with wild-type SIRT6 [24]. These variants suggest the possibility that superior SIRT6 activity contributes to human longevity and protection against malignancy. Given the established link between defective DNA repair and tumorigenesis, further studies are warranted to elucidate how SIRT6 dysfunction contributes to specific cancer types, and whether pharmacological activation of SIRT6 can be translated into clinically meaningful strategies for cancer prevention and therapy.

### 1.2. SIRT6 Is a Central Regulator of Metabolic Reprogramming

Another distinctive feature of SIRT6 is its multifaceted role in shaping cellular energy metabolism, spanning glucose utilization, mitochondrial function, and glycolytic reprogramming (Figure 1). Unlike sirtuins with narrow metabolic roles, SIRT6 exerts broad control over glycolysis, oxidative phosphorylation, and lactate secretion, which links its activity to both cancer metabolism and systemic metabolic diseases [12].

SIRT6 serves as a pivotal integrator of metabolic control and mitochondrial health. Its activation ameliorates mitochondrial dysfunction in doxorubicin-induced cardiomyopathy via Nrf2/FUNDC1 signaling [25], while in skeletal muscle, SIRT6 promotes oxidative reprogramming by suppressing Sox6 through CREB-dependent transcriptional activation, thereby enhancing mitochondrial capacity and muscle endurance [26]. This fundamental role in sustaining metabolic homeostasis and oxidative metabolism underpins its function as a tumor suppressor. Specifically, SIRT6 frequently restrains oncogenic glycolysis in cancer cells. Its deficiency induces metabolic reprogramming and thereby drives aggressive progression in various malignancies, including bladder cancer, breast cancer, and hepatocellular carcinoma [27,28,29].

Beyond cancer, a similar context-dependent metabolic switch orchestrated by SIRT6 is observed in non-malignant pathologies. For instance, under pathological settings such as type 2 diabetes mellitus, SIRT6 translocates from the nucleus to the cytoplasm, where it interacts with ENO3 to drive phosphoenolpyruvate accumulation and glycolytic flux, ultimately aggravating diabetic angiopathy [30]. Furthermore, silencing of VDAC1 alters SIRT6-related epigenetic and metabolic networks to rewire tumor metabolism [31]. These studies demonstrate that SIRT6 not only dictates glycolysis but also synchronizes mitochondrial and epigenetic programs essential for cellular fitness.

In brief, SIRT6 functions as a metabolic rheostat, toggling between oxidative and glycolytic programs depending on physiological context. Its loss or mislocalization promotes aerobic glycolysis, lactate accumulation, and tumorigenic potential, while its stabilization and activation favor oxidative metabolism and genome protection. In view of the established role of metabolic reprogramming as a cancer hallmark, targeting the SIRT6 axis offers a promising strategy for modulating energy homeostasis in both malignant and metabolic diseases.

### 1.3. SIRT6 Is a Master Epigenetic Regulator of Cancer Progression

SIRT6 is a nuclear NAD^+^-dependent deacylase that exerts profound effects on chromatin remodeling, histone modification, and transcriptional regulation, thereby linking epigenetic control to genome stability and disease progression. Unlike other sirtuins, SIRT6 shows high efficiency in deacetylating histone H3 at lysines 9, 18, and 56 (H3K9ac, H3K18ac, H3K56ac), which are epigenetic marks central to transcriptional activation [32].

SIRT6 regulates multiple chromatin-based processes relevant to cancer. First and foremost, it epigenetically represses oncogenic signaling by binding to promoter regions of target genes. For instance, in pancreatic cancer, SIRT6 facilitates the migration of pancreatic cancer cells and cytokine production by mediating deacetylation of histone H3K9 [33]. Subsequently, SIRT6 governs DNA repair through deacetylation of both histone and non-histone proteins, which coordinates base excision repair, double-strand break repair, and telomere maintenance. Loss of SIRT6 thus accelerates accumulation of mutations and chromosomal instability, both hallmarks of malignant transformation.

Moreover, SIRT6 modifies non-histone proteins critical for redox balance and stress response. It deacetylates and regulates transcription factors such as NF-κB, HIF-1α, and Nrf2, thereby coupling metabolic cues to inflammatory and antioxidant gene expression [34,35]. This function is of particular importance in cancer cells, where SIRT6 can act either as a tumor suppressor or, contextually, a tumor promoter depending on its control of glycolysis, oxidative stress, and apoptosis.

Overall, SIRT6 functions as a master epigenetic regulator, bridging chromatin dynamics, DNA repair, and transcriptional control with metabolic and stress pathways (Figure 1). Aberrations in SIRT6 activity can drive tumorigenesis through genomic instability, deregulated metabolism, and failure of transcriptional silencing.

## 2. The Dual Roles of SIRT6 in Different Cancer Types

SIRT6 exhibits a context-dependent functional duality in cancer, acting as either a tumor suppressor or an oncogene across different malignancies. The following part details the specific mechanisms underlying these divergent roles, as revealed by existing evidence (Table 1).

### 2.1. SIRT6 in Non-Small Cell Lung Cancer (NSCLC)

SIRT6 expression in NSCLC tissues and cell lines shows contradictory patterns. Some studies indicate that SIRT6 is lowly expressed in NSCLC, and its overexpression is associated with inhibited tumor progression [76,77,78]; however, other research suggests that SIRT6 is highly expressed in certain NSCLC cell lines [79], and high SIRT6 expression correlates with poor prognosis in lung adenocarcinoma, especially in patients with EGFR-mutated NSCLC [80,81].

**Figure 2:**
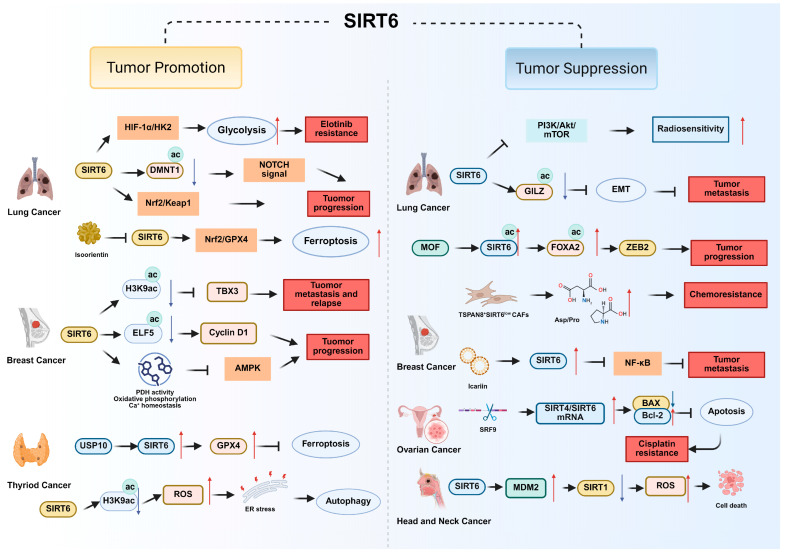
The tumor-promoting and tumor-suppressive molecular mechanism of SIRT6 in lung cancer, breast cancer, thyroid cancer, ovarian cancer and head and neck cancer.

SIRT6 enhances glycolysis through the HIF-1α/HK2 signaling axis, thereby promoting erlotinib resistance [36], while its suppression sensitizes cells to ferroptosis by regulating the SIRT6/Nrf2/GPX4 pathway [37]. Conversely, SIRT6 also exhibits tumor-suppressive properties in certain contexts. SIRT6 increases radiosensitivity by inhibiting PI3K/Akt/mTOR signaling [39] and stabilizes GILZ to suppress epithelial–mesenchymal transition and metastasis [40]. MOF-mediated acetylation of SIRT6 at K128, K160, and K267 reduces its deacetylase activity, impairs its interaction with FOXA2, activates ZEB2 transcription, and promotes NSCLC progression [41]. Pharmacological modulation of SIRT6 has shown therapeutic promise, with activators such as MDL-800 inhibiting proliferation and enhancing EGFR-TKI efficacy [81], while natural compounds like α-hederin exert anti-tumor effects through SIRT6-dependent glycolytic inhibition [76]. In addition, genetic silencing or small-molecule inhibition of SIRT6 induces oxidative stress-mediated apoptosis via the Nrf2/Keap1 pathway [34] and suppresses NOTCH signaling through the acetylation of DNMT1 (Figure 2 and Table 1) [38].

Collectively, these findings highlight SIRT6 as a double-edged regulator in NSCLC, functioning as either an oncogene or tumor suppressor depending on the cellular context, and underscore its potential as both a prognostic biomarker and a therapeutic target.

### 2.2. SIRT6 in Breast Cancer

Breast cancer represents a heterogeneous malignancy with diverse molecular subtypes and clinical outcomes [82]. Accumulating evidence highlights SIRT6 as a critical regulator of breast cancer pathogenesis, exhibiting context-dependent roles across different subtypes [83,84,85].

In HER2-positive tumors, the overexpression of SIRT6 promotes metastasis and relapse by repressing TBX3 through H3K9ac deacetylation, which confers cancer stem cell–like traits, dormancy, and poor relapse-free survival [42]. In contrast, in triple-negative breast cancer, upregulation of SIRT6 by icariin suppresses NF-κB/EMT signaling, induces redox-mediated apoptosis, and enhances anti-tumor immunity, which underscores its tumor-suppressive potential [45].

Within the tumor microenvironment, loss of SIRT6 in TSPAN8^+^ cancer-associated fibroblasts (CAFs) drives a senescence-like phenotype, metabolic reprogramming, and secretion of pro-tumorigenic metabolites that foster chemoresistance. Pharmacological activation of SIRT6, for instance with MDL-800, has been shown to counteract these effects [46]. At the metabolic level, SIRT6 promotes mammary tumorigenesis by enhancing pyruvate dehydrogenase activity, oxidative phosphorylation, and intracellular calcium homeostasis, while concurrently suppressing AMPK activation [43]. This metabolic rewiring sustains tumor growth and survival, demonstrating SIRT6 as a driver of bioenergetic adaptation.

Post-translational regulation of SIRT6 also impacts treatment responsiveness. AKT1-mediated phosphorylation promotes MDM2-dependent ubiquitination and degradation of SIRT6, thereby contributing to trastuzumab resistance in HER2-positive breast cancer [86]. Conversely, stabilization of SIRT6 restores drug sensitivity. Moreover, SIRT6-mediated deacetylation of ELF5 stabilizes this transcription factor and modulates CCND1 [44], suggesting an additional tumor-promoting axis (Figure 2 and Table 1). Clinical and meta-analytic evidence indicates that high SIRT6 expression generally associates with worse overall survival, though its effects vary across molecular subtypes [87]. Collectively, these findings underscore the dual tumor-promoting and tumor-suppressive functions of SIRT6 in breast cancer and suggest that therapeutic strategies must consider tumor subtype and context.

### 2.3. SIRT6 in Ovarian Cancer (OC)

Ovarian cancer remains one of the most lethal gynecologic malignancies, largely due to the development of chemoresistance and late-stage detection. Cisplatin-induced extracellular vesicles carrying the lncRNA PANDAR have been shown to promote chemoresistance and aggressiveness of OC cells through the SRSF9-SIRT4/SIRT6 axis. Specifically, PANDAR recruits SRSF9 to nuclear bodies under cisplatin stress, suppressing apoptosis and altering the ratio of SIRT4/SIRT6 transcripts, thereby facilitating survival and adaptation of OC cells in a hostile microenvironment (Figure 2 and Table 1). This demonstrates that SIRT6 is part of a stress-adaptive network contributing to chemoresistant phenotypes [47].

### 2.4. SIRT6 in Head and Neck Cancer (HNSCC)

HNSCC is a highly aggressive malignancy characterized by metabolic reprogramming and resistance to cell death [88]. Recent studies demonstrates that SIRT6 is an important tumor suppressor in this context [89]. SIRT6 promotes cell death in HNSCC through a unique interplay with SIRT1. Specifically, SIRT6 induces MDM2-mediated degradation of SIRT1, thereby relieving the protective role of SIRT1 and allowing accumulation of reactive oxygen species (ROS), which ultimately drive cancer cell death [48] (Figure 2 and Table 1). This pathway emphasizes the functional divergence within the sirtuin family and underscores the pro-apoptotic role of SIRT6 in HNSCC.

### 2.5. SIRT6 in Thyroid Cancer

SIRT6 plays a predominantly oncogenic role in thyroid cancer. In papillary thyroid cancer, SIRT6 promotes tumor progression and epithelial–mesenchymal transition. Specifically, SIRT6 overexpression depletes H3K56ac at the NRROS locus, increasing ROS production and triggering ER stress–induced autophagy. This autophagy mediates degradation of GLUT1, thereby modulating the Warburg effect and altering tumor metabolism. In addition, SIRT6-driven metabolic reprogramming is confirmed by ^18^F-FDG PET/CT in xenografts, which highlights its relevance to tumor energy dynamics [49]. SIRT6 is also linked to ferroptosis resistance in thyroid carcinoma. SIRT6 upregulated by USP10 stabilizes GPX4, thereby attenuating erastin-induced ferroptosis and enhancing tumor growth, migration, and invasion [50] (Figure 2 and Table 1).

### 2.6. SIRT6 in Pancreatic Cancer

Pancreatic ductal adenocarcinoma (PDAC) remains one of the most aggressive malignancies with limited therapeutic options and poor prognosis [90]. SIRT6 has been implicated in the regulation of pancreatic ductal adenocarcinoma (PDAC) progression, particularly by promoting tumor cell migration and metastatic dissemination.

Elevated SIRT6 expression enhances epithelial–mesenchymal transition (EMT) and fosters invasion [91], positioning it as a potential pro-metastatic factor in this malignancy. Consistent with this view, multiple selective SIRT6 inhibitors, including allosteric modulators such as compound 11e and JYQ-42, as well as pyrrole-pyridinimidazole derivative 8a, have shown potent suppression of pancreatic cancer cell proliferation, invasion, and liver metastasis in vitro and in vivo [33,92]. Emerging evidence further indicates that SIRT6 contributes to therapeutic resistance in PDAC. In contrast to lung cancer, SIRT6 inhibition enhances gemcitabine sensitivity through modulation of the PI3K/AKT/mTOR and ERK signaling pathways [51]. Moreover, 8a exerts additional anti-angiogenic effects by downregulating VEGF, HIF-1α, and p-VEGFR2, further supporting its therapeutic promise [52].

**Figure 3:**
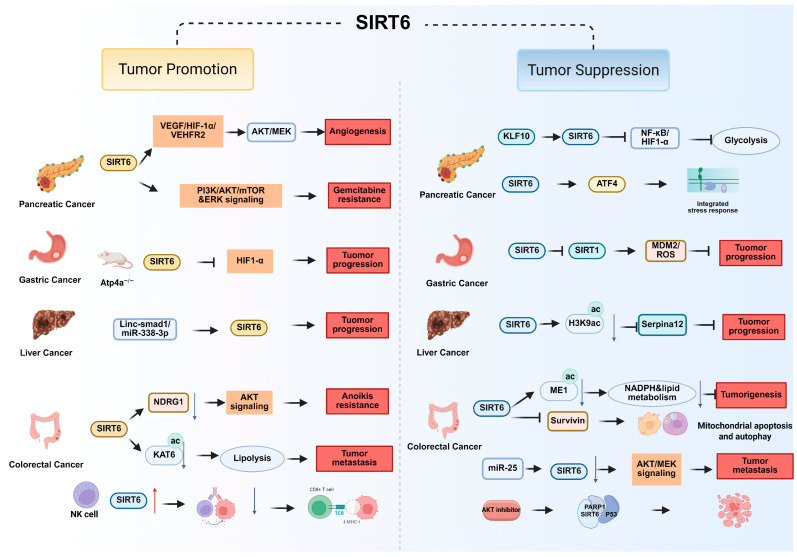
The tumor-promoting and tumor-suppressive molecular mechanism of SIRT6 in digestive cancers.

Paradoxically, SIRT6 may also act as a tumor suppressor under specific conditions. In KLF10-deficient PDAC, SIRT6 upregulation reverses EMT, inhibits glycolysis, and reduces distant metastasis by regulating NF-κB and HIF-1α signaling [53]. Restoration or pharmacological induction of SIRT6 in this context prolongs survival in preclinical models. These seemingly contradictory findings suggest a context-dependent duality, whereby SIRT6 may either promote or suppress PDAC progression depending on molecular background and tumor subtype. In addition, SIRT6 expression was shown to regulate the integrated stress response (ISR) via ATF4 stabilization (Figure 3 and Table 1). In mouse model, loss of SIRT6 defines the basal PDAC subtype, which displays heightened vulnerability to transcriptional CDK7/CDK9 inhibition [54], revealing an exploitable therapeutic window for this subgroup.

In summary, these findings illustrate the dual nature of SIRT6 in pancreatic cancer: acting as a promoter of metastasis and chemoresistance in some contexts, while functioning as a suppressor of EMT, glycolysis, and tumor progression in others. Such complexity underscores the necessity for precision medicine approaches when targeting SIRT6, with therapeutic strategies tailored to tumor subtype, molecular background, and treatment regimen.

### 2.7. SIRT6 in Gastric Cancer

SIRT6 plays a multifaceted role in gastric cancer (GC), exerting both tumor-suppressive and context-dependent oncogenic functions. SIRT6 overexpression suppresses SIRT1, leading to elevated MDM2 and ROS levels that drive gastric cancer cell death and reduce xenograft tumor growth [56]. Transcriptomic analyses of the gastric inflammation-carcinoma sequence reveal stage-specific SIRT6 activity, with peak expression during intestinal metaplasia and evidence of tumor-suppressive effects through inhibition of cell proliferation and migration [77,93]. SIRT6 also modulates tumor-associated processes indirectly by interacting with autophagy regulators such as ATG10 suggests a potential inhibitory role in GC progression [87,94]. Nevertheless, SIRT6 expression is upregulated alongside PI3K/AKT/mTOR signaling and the Warburg effect which links it to metabolic reprogramming and gastric mucosal transformation in mouse models of parietal cell atrophy [55] (Figure 3 and Table 1). Moreover, immune-related studies highlight reduced SIRT6 expression in dysfunctional NK cells in gastric cancer, implicating it in impaired antitumor immunity [62].

### 2.8. SIRT6 in Liver Cancer

Hepatocellular carcinoma (HCC) represents the most common form of primary liver cancer and remains a major cause of cancer-related mortality worldwide. Increasing evidence highlights SIRT6 as a critical factor in HCC progression, with functions spanning metabolic regulation, genomic stability, and therapy response [95,96].

SIRT6 suppresses the transcription of Serpina12 through histone H3K9 deacetylation at its promoter, thereby reducing glucose uptake, lactate production, and aberrant lipid synthesis, ultimately limiting cancer cell proliferation and survival [59]. In line with its tumor-suppressive role, hepatic-specific SIRT6 knockout mice display increased susceptibility to both spontaneous and chemically induced liver tumors [97]. Stabilization of SIRT6 by USP48 prevents glycolytic shift and tumor progression [29], whereas dysregulation of non-coding RNAs, including Linc-smad7 and miR-338-3p, disrupts SIRT6 expression to promote proliferation, invasion, and chemoresistance [57,58] (Figure 3 and Table 1). In addition, SIRT6 contributes to the maintenance of genomic stability. It facilitates DNA double-strand break repair and mitigates oxidative stress, thereby preserving chromatin integrity and reducing the accumulation of DNA damage. Mechanistic investigations demonstrate that SIRT6 safeguards DNA integrity by regulating chromatin accessibility at sites of damage, and recent PROTAC-based studies show that pharmacological depletion of SIRT6 impairs DNA repair capacity, sensitizing HCC cells to radiotherapy and kinase inhibitors [98].

In view of the above evidence, SIRT6 emerges as a pivotal safeguard against liver cancer. Its dual capacity to maintain hepatic metabolic homeostasis and to suppress tumor-promoting signaling cascades positions it as a promising therapeutic target.

### 2.9. SIRT6 in Colorectal Cancer

SIRT6 displays a complex role in colorectal cancer (CRC), functioning in both tumor-suppressive and tumor-promoting capacities depending on cellular and microenvironmental contexts. On one hand, SIRT6 supports anoikis resistance by repressing NDRG1, which enhances AKT signaling and promotes metastatic potential [60]. SIRT6-mediated deacetylation also modulates lipolysis through KAT8 regulation, impacting the invasive and migratory properties of CRC cells [61]. Moreover, tumor-derived extracellular vesicles delivering miR-25 inhibit SIRT6, thereby promoting metastasis via the Lin28b/NRP-1 axis [65].

On the tumor-suppressive side, SIRT6 directly represses Survivin transcription, triggering mitochondrial apoptosis and autophagy in colon cancer [66], and cooperates with p53/PARP1 to induce parthanatos upon AKT inhibition [67]. SIRT6 counteracts ACAT1-driven ME1 acetylation to suppress NADPH generation and lipid metabolism, thereby restraining tumorigenesis [64]. Pharmacological activation of SIRT6 using small-molecule activators (e.g., MDL-811) exerts broad anti-proliferative effects and synergizes with vitamin D3 therapy [99], while dietary interventions such as methionine restriction enhance PD-1 blockade efficacy by relieving SIRT6 suppression [100]. SIRT6 is also upregulated in exhausted NK cells within CRC tissue and dampens their cytotoxicity [62,63] (Figure 3 and Table 1). Clinical analyses further reveal a context-dependent role: reduced SIRT6 expression predicts favorable prognosis in several solid tumors, yet poor outcome in gastrointestinal cancers [87]. Collectively, these findings underscore the dual nature of SIRT6 in CRC—acting as a metabolic and epigenetic regulator that can either suppress or facilitate tumor progression.

### 2.10. SIRT6 in Malignant Tumors of the Urinary System

Among urological malignancies, prostate cancer, renal cell carcinoma, and bladder cancer are the most common. Prostate cancer typically arises in a small region of the prostate and generally exhibits slow growth [101]. Renal cell carcinoma is a malignant tumor originating in the renal parenchyma, while the most common type of bladder cancer is urothelial carcinoma [102]. Globally, the incidence of urological cancers is increasing, with prostate cancer showing particularly rapid growth. The incidence in men is generally higher than in women, which may be related to greater exposure to carcinogenic factors. These cancers predominantly affect individuals over 40 years of age, with risk increasing with age.

**Figure 4:**
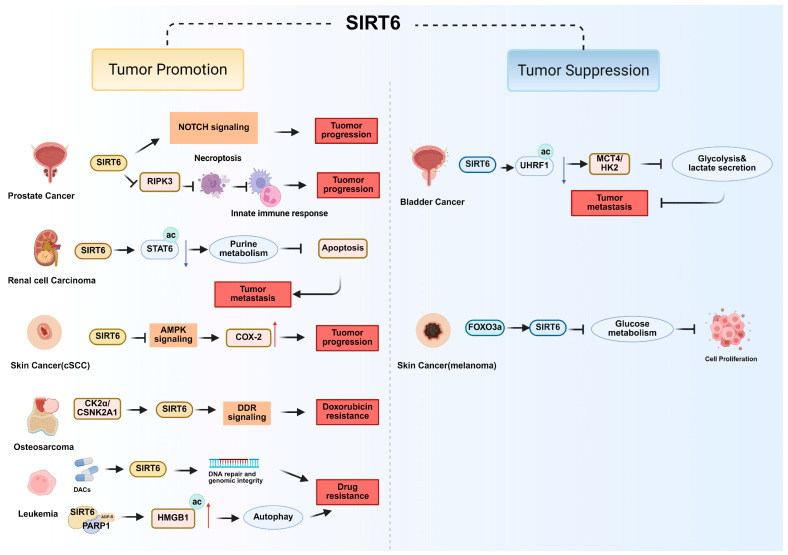
The tumor-promoting and tumor-suppressive molecular mechanism of SIRT6 in urinary cancers, skin cancer, osteosarcoma and leukemia.

SIRT6 exerts diverse and context-dependent functions in urinary system cancers. It acts predominantly as a tumor suppressor in bladder and kidney malignancies but as a tumor promoter in prostate cancer [80,103]. SIRT6 has emerged as a critical tumor suppressor in bladder cancer. Clinical analyses demonstrate that SIRT6 expression is frequently downregulated in bladder cancer and correlates with poor overall survival [104]. SIRT6 interacts with and deacetylates UHRF1, promoting its ubiquitination and degradation via β-TrCP1. SIRT6 deficiency stabilizes UHRF1, which in turn enhances glycolysis and lactate secretion by upregulating MCT4 and HK2 expression, thereby driving tumor proliferation, migration, and self-renewal [28] (Figure 4 and Table 1). Pharmacological inhibition of UHRF1 markedly attenuates the malignant progression of SIRT6-deficient BLCA, suggesting that targeting the SIRT6-UHRF-glycolysis axis represents a novel therapeutic vulnerability.

In renal clear cell carcinoma, SIRT6 deacetylates STAT6 at lysine 284, thereby modulating the AF9/STAT6 signaling axis that governs purine metabolism and apoptosis [68] (Figure 4 and Table 1). Loss of this regulatory pathway diminishes apoptosis and enhances invasive migration, promoting metastatic spread.

By contrast, in prostate cancer, SIRT6 expression is elevated and correlates with advanced stage, higher Gleason scores, and reduced survival. SIRT6 promotes tumorigenesis by activating oncogenic pathways such as Notch signaling, while also inhibiting necroptosis-mediated immune responses through repression of RIPK3, thereby enabling tumor immune evasion [69] (Figure 4 and Table 1). Importantly, targeted silencing of SIRT6 via engineered exosomes carrying siRNA significantly impairs tumor growth and metastasis in preclinical models, underscoring its therapeutic relevance [70].

To summarize, these findings indicate that SIRT6 plays a dualistic role in urinary system cancers, with tumor-suppressive effects in bladder and kidney cancers and tumor-promoting activity in prostate cancer.

### 2.11. SIRT6 in Skin Cancer

SIRT6 exerts context-dependent roles in skin cancer, acting as both an oncogenic factor and a tumor suppressor [105]. In cutaneous squamous cell carcinoma (cSCC), SIRT6 is frequently overexpressed and associated with enhanced proliferation, migration, and epithelial–mesenchymal transition (EMT). Silencing or pharmacological inhibition of SIRT6 suppresses cSCC progression, promoting differentiation, blocking cell cycle progression, and reducing EMT markers, suggesting that SIRT6 supports tumor propagation [71]. In addition, SIRT6 deletion abrogates DMBA/TPA-induced tumorigenesis in mice, indicating its critical role in cSCC initiation [106] (Figure 4 and Table 1).

By contrast, SIRT6 appears to have tumor-suppressive functions in melanoma. The FOXO3a-SIRT6 axis represses glycolysis, thereby restraining melanoma growth [72] (Figure 4 and Table 1). Loss-of-function SIRT6 mutations from melanoma patients compromise DNA repair capacity, destabilize the genome and contribute to tumorigenesis [107].

### 2.12. SIRT6 in Osteosarcoma

SIRT6 has been implicated as a critical regulator of chemoresistance in osteosarcoma. Clinical studies revealed that high SIRT6 expression is significantly associated with shorter overall and relapse-free survival, particularly in patients receiving adjuvant chemotherapy [108]. SIRT6 promotes activation of the DNA damage repair (DDR) pathway, thereby attenuating the cytotoxic effects of doxorubicin. Knockdown of SIRT6 enhances doxorubicin-induced apoptosis, whereas overexpression confers resistance. Upstream regulation of SIRT6 occurs through phosphorylation at Ser338 by casein kinase 2α (CK2α/CSNK2A1) (Figure 4 and Table 1). This post-translational modification further activates DDR signaling, strengthening chemoresistance. Notably, pharmacological blockade of SIRT6 or inhibition of CK2α (e.g., with emodin) synergistically enhances the anti-tumor activity of doxorubicin in osteosarcoma models [73]. These findings suggest that targeting the CK2α-SIRT6-DDR axis may represent a promising therapeutic strategy to overcome chemoresistance in osteosarcoma.

### 2.13. SIRT6 in Leukemia

Recent evidence has shown a functional link between SIRT6 and therapeutic response in leukemia [74]. SIRT6 activates the PARP1-HMGB1-autophagy pathway and induces chemotherapy resistance in leukemia [75]. Moreover, FDA-approved DNA hypomethylating agents (DHAs) such as decitabine (DAC) and 5-azacytidine (5AC), widely used in acute myeloid leukemia (AML), have been shown to selectively activate SIRT6 enzymatic activity without significantly affecting other sirtuin isoforms. SIRT6 activation by DHAs appears to support genomic integrity and DNA repair, providing a protective role in leukemia cells under epigenetic therapy (Figure 4 and Table 1). Clinically, this finding reveals that the combination of nucleoside analog DHAs with SIRT6 inhibitors or DNA-damaging agents may be counterproductive [74], as SIRT6 activity could mitigate therapeutic efficacy by stabilizing genome maintenance processes.

## 3. Conclusions

SIRT6 exemplifies a paradigm of context-dependent functionality within the sirtuin family, operating as both a gatekeeper of genomic integrity and a modulator of oncogenic metabolism. Its tumor-suppressive attributes are frequently mediated through the preservation of DNA repair fidelity and the negative regulation of glycolytic pathways, thereby constraining neoplastic transformation. Conversely, in established malignancies, SIRT6 can be co-opted to fuel tumor adaptation by rewiring energy metabolism and sustaining survival under therapeutic stress.

This functional duality is not merely organ-specific but is often dictated by the molecular landscape of individual tumors, including the status of key oncogenic drivers and tumor suppressor networks. The metabolic flexibility of SIRT6 enables it to interface with nutrient-sensing pathways and redox homeostasis, thereby positioning it as a critical adaptive node in tumor evolution. Furthermore, its non-canonical roles in modulating immune cell function within the tumor microenvironment add another layer of complexity to its therapeutic targeting.

Specifically, SIRT6 possesses distinctive structural features that enable unique protein–protein interactions and substrate specificity compared to other sirtuins. Its capacity to integrate metabolic signals with chromatin remodeling and transcriptional output provides a mechanistic basis for its pleiotropic effects on tumor behavior. The recent identification of allosteric regulatory sites and tissue-specific post-translational modifications offers new avenues for selective pharmacological intervention.

The expanding repertoire of SIRT6 functions underscores its potential as a multi-faceted therapeutic target. Future research directions should focus on delineating the precise molecular determinants that govern its context-dependent switching between tumor-promoting and suppressive states. Developing subtype-specific SIRT6 modulators, combined with advanced biomarker-driven patient stratification, may unlock novel combinatorial regimens for precision oncology aimed at cancer prevention, treatment optimization, and metastasis suppression. Ultimately, harnessing the dual nature of SIRT6 requires mapping its complex interactome across the continuum of malignant progression.

## Figures and Tables

**Table 1:** The specific mechanism of SIRT6 in diverse cancers.

Cancer Type	Function of SIRT6	Pathways and Molecular Mechanism	References
Non-Small Cell Lung Cancer (NSCLC)	Tumor promotion	SIRT6 promotes erlotinib resistance by enhancing glycolysis through the HIF-1α/HK2 signaling axis.	[36]
		SIRT6 Sensitizes cells to ferroptosis by regulating the SIRT6/Nrf2/GPX4 pathway.	[37]
		SIRT6 induces oxidative stress-mediated apoptosis via the Nrf2/Keap1 pathway.	[34]
		SIRT6 activates NOTCH signaling through DNMT1 acetylation to promote tumor progression.	[38]
	Tumor suppression	SIRT6 inhibits PI3K/Akt/mTOR signaling to increase radiosensitivity	[39]
		SIRT6 deacetylates GILZ to suppress EMT and metastasis.	[40]
		SIRT6 deacetylates FOXA2 to inhibit ZEB2 transcription that suppress tumor growth.	[41]
Breast Cancer	Tumor promotion	SIRT6 overexpression promotes metastasis and relapse by repressing TBX3 through H3K9ac deacetylation.	[42]
		SIRT6 enhances PDH activity, oxidative phosphorylation and Ca^2+^ homeostasis to inhibit AMPK signaling that suppresses tumor growth.	[43]
		SIRT6 deacetylates and stabilizes ELF5 to upregulate cyclin D1 (CCND1) and promote cell proliferation.	[44]
	Tumor suppression	SIRT6 upregulated by icariin suppresses NF-κB/EMT signaling, induces redox-mediated apoptosis and suppresses tumor metastasis.	[45]
		Loss of SIRT6 in TSPAN8^+^ CAFs induces chemoresistance by promoting metabolic reprogramming and secretion of pro-tumorigenic metabolites.	[46]
Ovarian Cancer	Tumor suppression	SIRT4/SIRT6 mRNA level increases BAX/Bcl-2 expression level and induces tumor cell apoptosis, sensitizing cells to cisplatin.	[47]
Head and Neck Cancer	Tumor suppression	SIRT6 induces MDM2-mediated degradation of SIRT1 and accumulates of ROS, driving cancer cell death.	[48]
Thyroid Cancer	Tumor promotion	SIRT6 accumulates ROS then activates endoplasmic reticulum stress (ER stress) and subsequently induced autophagy by deacetylating H3K56ac	[49]
		SIRT6 elevated by USP10 attenuates the ferroptosis to promote thyroid cancer malignancy by facilitating GPX4	[50]
Pancreatic Cancer (PDAC)	Tumor promotion	SIRT6 activates PI3K/AKT/mTOR and ERK signaling pathways to induce gemcitabine resistance	[51]
		SIRT6 upregulates VEGF/HIF-1α/VEGFR2 signaling and activates AKT/MEK signaling to promote angiogenesis	[52]
	Tumor suppression	KLF10-SIRT6 reverses EMT, suppresses glycolysis and reduces metastasis via NF-κB & HIF-1α.	[53]
		SIRT6 regulates ISR via ATF4 and loss of SIRT6 defines basal subtype vulnerable to CDK7/9 inhibition.	[54]
Gastric Cancer	Tumor promotion	SIRT6 inhibits HIF1-α to adapt to the microenvironment of cell hypoxia in in Atp4a−/− mice.	[55]
	Tumor suppression	SIRT6 suppresses SIRT1 to upregulate MDM2 and ROS expression, suppressing tumor progression.	[56]
Liver Cancer (HCC)	Tumor promotion	SIRT6 is upregulated by Linc-smad7 and miR-338-3p and promotes cancer progression.	[57,58]
	Tumor suppression	SIRT6 suppresses Serpina12 expression through histone H3K9 deacetylation and limits cancer progression.	[59]
Colorectal Cancer (CRC)	Tumor promotion	SIRT6 inhibits NDRG1 repression and activates AKT signaling to support anoikis resistance.	[60]
		SIRT6 deacetylates KAT6 to modulate lipolysis and promote CRC metastasis.	[61]
		SIRT6 is upregulated in CRC-infiltrating NK cells and decreases cytotoxicity and immune evasion.	[62,63]
	Tumor suppression	SIRT6 counteracts ACAT1-driven ME1 acetylation and downregulates nicotinamide adenine dinucleotide phosphate (NADPH) & lipid metabolism, thereby restraining tumorigenesis.	[64]
		MiR-25 downregulates SIRT6 to activate Lin28b/NRP-1 axis and promote tumor metastasis.	[65]
		SIRT6 represses Survivin to induce mitochondrial apoptosis and autophagy.	[66]
		SIRT6 cooperates with p53/PARP1 to promote the formation of PAR polymer and cell death upon AKT inhibition.	[67]
Bladder Cancer	Tumor suppression	SIRT6 deacetylates UHRF1 and upregulates MCT4 and HK2 expression, in turn inhibiting glycolysis, lactate secretion and tumor migration.	[28]
Renal Cell Carcinoma	Tumor suppression	SIRT6 deacetylates STAT6 and modulates the AF9/STAT6 signaling axis that inhibits purine metabolism and promotes apoptosis.	[68]
Prostate Cancer	Tumor promotion	SIRT6 inhibits necroptosis-mediated immune responses through repression of RIPK3.	[69]
		SIRT6 promotes tumorigenesis by activating Notch signaling	[70]
Skin Cancer	Tumor promotion	SIRT6 inhibits AMPK pathway and regulates COX-2 stability to promote cutaneous squamous cell carcinoma (cSCC) progression.	[71]
	Tumor suppression	SIRT6 regulating by FOXO3a inhibited glucose metabolism and tumor cell proliferation in melanoma.	[72]
Osteosarcoma	Tumor promotion	SIRT6 regulated by CK2α activates the DNA damage repair (DDR) pathway and enhances doxorubicin resistance.	[73]
Leukemia	Tumor promotion	SIRT6 activated by DACs supports genomic integrity and DNA repair and protects leukemia cells.	[74]
		SIRT6 catalyzes the monoADP-ribosylation of PARP1 and acetylates HMGB1 that promoting autophagy and drug resistance	[75]

## Data Availability

No new data were created or analyzed in this study.
